# Interactions between Innate Immunity, Microbiota, and Probiotics

**DOI:** 10.1155/2015/501361

**Published:** 2015-05-18

**Authors:** GianMarco Giorgetti, Giovanni Brandimarte, Federica Fabiocchi, Salvatore Ricci, Paolo Flamini, Giancarlo Sandri, Maria Cristina Trotta, Walter Elisei, Antonio Penna, Piera Giuseppina Lecca, Marcello Picchio, Antonio Tursi

**Affiliations:** ^1^Digestive Endoscopy & Nutrition Unit, “S. Eugenio” Hospital, Piazzale dell'Umanesimo 10, 00144 Rome, Italy; ^2^Division of Internal Medicine and Gastroenterology, “Cristo Re” Hospital, Via delle Calasanziane 25, 00167 Rome, Italy; ^3^Clinical Nutrition Unit, “S. Eugenio” Hospital, Piazzale dell'Umanesimo 10, 00144 Rome, Italy; ^4^Clinical Nutrition Unit, “Umberto I” Hospital, Viale del Policliniuco 185, 00186 Rome, Italy; ^5^Division of Internal Medicine, “S. Eugenio” Hospital, Piazzale dell'Umanesimo 10, 00144 Rome, Italy; ^6^Division of Gastroenterology, ASL Roma H, Via Borgo Garibaldi 12, Albano Laziale, 00041 Rome, Italy; ^7^Division of Gastroenterology, “S. Paolo” Hospital, Contrada Capo Scardicchio 82, 00123 Bari, Italy; ^8^Division of General Surgery, “P. Colombo” Hospital, ASL Roma H, Via Orti Ginnetti 7, Velletri, 00049 Rome, Italy; ^9^Gastroenterology Service, ASL BAT, Via Torino 49, 76123 Andria, Italy

## Abstract

The term “microbiota” means genetic inheritance associated with microbiota, which is about 100 times larger than the guest. The tolerance of the resident bacterial flora is an important key element of immune cell function. A key role in the interaction between the host and the microbiota is played by Paneth cell, which is able to synthesize and secrete proteins and antimicrobial peptides, such as *α*/*β* defensins, cathelicidin, 14 *β*-glycosidases, C-type lectins, and ribonuclease, in response to various stimuli. Recent studies found probiotics able to preserve intestinal homeostasis by downmodulating the immune response and inducing the development of T regulatory cells. Specific probiotic strain, as well as probiotic-driven metabolic products called “postbiotics,” has been recently recognized and it is able to influence innate immunity. New therapeutic approaches based on probiotics are now available, and further treatments based on postbiotics will come in the future.

## 1. Introduction

The term “microbiota” means genetic inheritance associated with microbiota, which is about 100 times larger than the guest. The functions of the microbiota are many and until now it is considered a virtual organ of the human body [1].

Main functions of microbiota are amino acids and vitamins synthesis and energy extraction from nonabsorbable polysaccharides. It also contributes to the intestinal wall integrity, acting against pathogens and supporting immune system growth [1].

Assessing the human microbiota is one of main topics in biology and medicine regarding the relationships emerging between types of microbiota and development of important diseases. From an evolutionary point of view, we know that microbiota accompanied the evolution of the species for 400 million years, and the human race would probably never have existed without this close symbiosis. In fact the human microbiota gives the human race genes that human have progressively lost in their evolutionary cycle, creating a real superorganism made up of human and microbiotic characteristics. This superorganism has 10^14^ cells arising from microbiota and 10^13^ cells arising from the human race, a biological mass composed of 98% human material, but with 3^105^ genes arising from microbiota and 3^10^ genes from human genes [1]. Our microbiota begins immediately after birth, remaining relatively constant until adult age and reducing in old age. The “core” of intestinal microbiota remains constant and unmodified almost like our fingerprints. It is mainly represented by bacteria but is also composed of virus, fungus, and protozoa [1].

In the last 10 years the interest in intestinal microbiota has grown rapidly following molecular biology techniques that have overcome the limits of the old culture techniques. Microbiota lives in close contact with our intestine. Both intestine and microbiota form an ecological system that has many cellular and molecular components that work to maintain an adequate fast and efficient immune response which respects the morphological and functional integrity of the bowel.

## 2. Intestinal Microbiota

The identification of the resident intestinal bacterial species is mainly based on the analysis of 16S ribosomal RNA subunit obtained from the amplification by polymerase chain reaction of nucleic acids extracted from the lumen of the intestinal mucosa and faeces [1]. Using molecular biology we are able to overcome the relatively long period of growth of microorganisms and to isolate approximately 60–80% of the commensal bacterial species. The distribution of the microbiota along the digestive tract is not uniform: it is very low in the stomach (0–10%) due to the bactericidal action of the chlorhydric acid (real barrier in the entrance of many bacterial species in digestive tract), increases gradually in the small intestine (10^7^ × 10^8^), and reaches very high concentrations in the colon (10¹¹×10¹²) where the microbiota is represented mainly by Gram-negative anaerobic bacteria. Until now, between 1000 and 1150 bacterial species have been identified; among them every adult hosts about 160. Concentrations and bacterial biodiversity (composition) in different intestinal tracts depend on several factors. Some of these are intrinsic to the host, such as acid secretion, intestinal motility, and immune response, while others are exogenous such as diet, taking antibiotics, PPI, laxatives, and opioids. The microbiota changes at different stages of life. At birth, the gut is sterile, and it is colonized by the vaginal and intestinal microbiota of the mother or by the skin for those born by caesarean delivery. Intestinal microbiota changes with breastfeeding and weaning and in adulthood it remains stable but changes again in the elderly [2].

**Figure 1:**
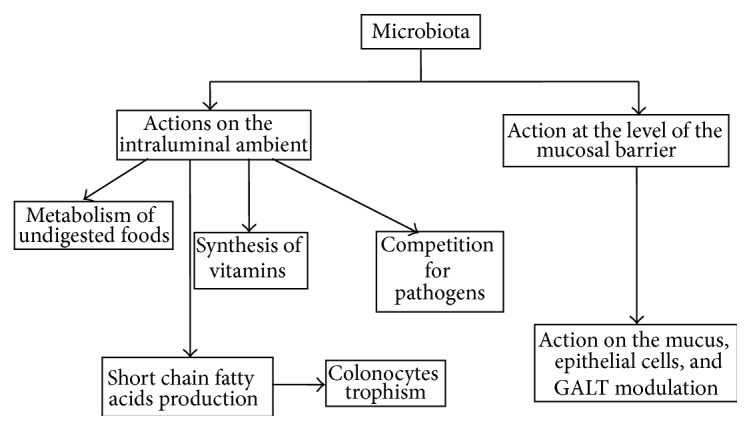
Action taken by microbiota in normal conditions.

It also plays an important role in metabolic activities in humans, considering that the microbiota biomass is comparable to that of the liver. Among the main functions are the synthesis of essential amino acids and vitamins (K, B_2_, B_1_, folic acid, biotin, and pantothenic acid) and extraction of energy from components in the diet as some are not digestible polysaccharides of plant origin. Moreover, it contributes to maintaining the integrity of the intestinal wall, modulating responses to pathogenic noxae, and representing a key factor in the maturation of the immune system. Although 50 bacterial* phyla* have been identified, just three are living in our colon: Firmicutes, Bacteroidetes, and Actinobacteria. Actions taken by microbiota in normal conditions are reported in Figure 1.

## 3. Mucosal Layer

Microbiota is separated from the epithelial cells by a network of glycans (glycocalyx and mucus layer) mainly produced by mucipare cells.

**Figure 2:**
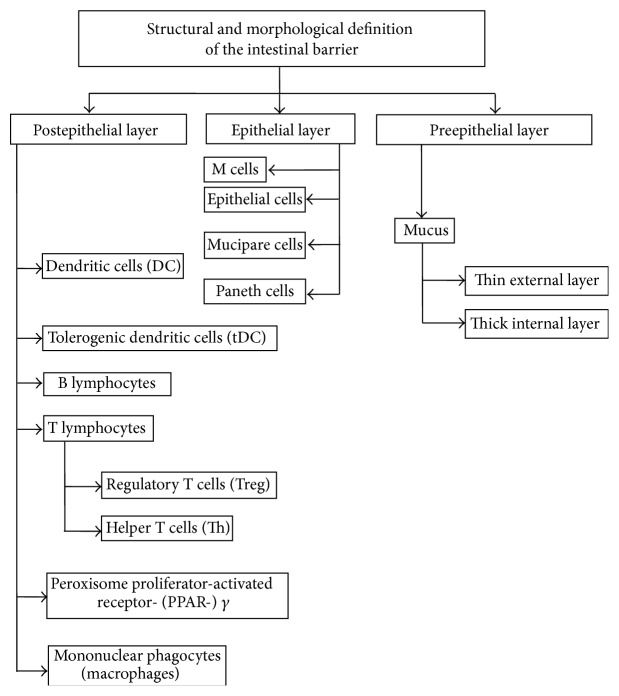
Structural and morphological definition of the intestinal barrier.

The intestinal epithelial barrier is composed of several layers of defense as follows (see Figure 2):

- the mucus that opposes static hindrance to the bacteria;
- the epithelial layer which is composed of absorptive enterocytes, goblet cells, Paneth cells, tuft and cup cells, M cells, and enterochromaffin cells;
- immune cells such as intraepithelial *γδ* and *αβ* lymphocytes, retinoic acid receptor-related orphan receptor (ROR)y*τ*+, lymphoid-tissue inductor (LTI), and NKP46T innate immune cells; others have direct access to the lumen-like antigen, presenting cells or neutrophils after infection.

Paneth cells are secretory cells specialized in the production of antimicrobial peptides (AMP). These include defensins, lisozymes, C-type lectins, and cathelicidins. RORy*τ*+ cells release IL-22, which is required for both epithelial cell repair and antibacterial activity.

The mucus layer is fundamental to guarantee the integrity of the intestinal wall. It is rapidly improved through an average daily production of 5 liters. Its main constituent is the mucin, a glycoprotein mainly represented by subtype 2 (MUC2) [2]. The mucus of the stomach and the colon is particularly thick and is made from two layers: an inner (50/200 *μ*m) compact and with small pores that prevent the physical penetration of bacteria [2] and one external (70–150 *μ*m) composed primarily of glycans. Both these layers contribute to the defensive effect. For example, mice with MUC2 deficiency spontaneously develop an inflammatory process located in the intestinal mucosa [3].

Only some bacteria are able to be located in the niche of the layer of mucus. This is because the microbiota develops some systems of adaptation such as the production of enzymes that degrade the mucus. This is an important factor for the survival of the bacteria in this habitat, since this activity allows the production of nutrients for the bacteria themselves. Microbiota also contributes to the production of mucus and the thickness of the mucus layer. This occurs also through the stimulation of the synthesis of mucin by some bacterial components such as lipopolysaccharide (LPS) and short chain fatty acids (SCFA) [4].

## 4. Interaction between Epithelium and Microbiota and Occurrence of the Intestinal Inflammation

The intestinal mucosa is a major constituent of the human immune system. In fact, small intestine contains about 1 × 10^10^ plasma cells per meter, approximately 80% of all plasma cells contained in the whole body. In addition, the daily production of IgA in the intestinal lumen is higher than the total daily production of IgG [1].

Considering that the intestinal lamina propria is infiltrated by lymphocytes, plasma cells, and macrophages, we can affirm that occurrence of inflammatory cells may represent a physiological phenomenon in the human intestine of human. This infiltration, called “physiological inflammation” of the normal intestinal mucosa, occurs and develops after the massive stimulation of the mucosal immune system by luminal antigens. In fact intestinal mucosa is constantly exposed to an enormous antigen load present in the lumen, by which the resident bacterial flora contributes predominant. This means that a range of structures and cells are responsible for the control and the maintenance of an adequate immune response in the normal intestinal mucosa and that a harmful immune response against exposure to luminal antigens is not happening. Due to the massive stimulation by luminal antigens, the immune cells that physiologically infiltrate the intestinal mucosa, which are mainly memory T cells, are both increased and activated and develop a local immune response [1]. The tolerance of the resident bacterial flora is therefore an important key element of immune cell function. In fact the excessive degradation of the mucin layer by bacteria facilitates the access of luminal antigens and then the activation of the immune response. For example, it has been shown that alterations in the mucus layer contribute to the pathogenesis of inflammatory bowel disease (IBD) [5]. If we compare the healthy people with IBD patients, we can observe a larger amount of bacteria in contact with the epithelial cells in IBD patients than in healthy controls. The reason why this occurs has been clarified. Although leukocyte reaction tries to contain the bacteria living in the bowel lumen, thanks to its antimicrobial activity exerting in the external layer, some bacteria reach the mucosa owing to impairment of mucosal layer causing inflammation [6]. This has been confirmed by Ganesh et al. These authors have demonstrated that, in an animal model of mice with microbiota consisting essentially of eight bacterial species, the presence of a commensal bacterium (*Akkermansia muciniphila*) is able to degrade the mucin and exacerbate the inflammation and the severity of symptoms due to* Salmonella enterica* Typhimurium infection [7].

## 5. Interaction between Innate Immunity, Paneth Cells, and Toll-Like Receptors

A key role in the interaction between the host and the microbiota is played by Paneth cell. These are specialized typical epithelial cells of the small intestine but may be detected in lower concentrations in the stomach and colon. The Paneth cells are able to synthesize and secrete proteins and antimicrobial peptides, such as *α*/*β* defensins, cathelicidin, 14 *β*-glycosidases, C-type lectins, and ribonuclease, in response to various stimuli, including components of the bacterial surface and toll-like receptors (TLR) agonists [8, 9]. Defensin 5 (HD-5) is also produced by the Paneth cells, playing a role both in protection against pathogens and in determining the composition of the microbiota [10]. In particular, this second activity works by controlling the number of microorganisms, in this way contributing to host defense. In fact the transgenic mice for the HD-5 indeed show a greater resistance to oral* Salmonella* Typhimurium [11].

The epithelial cells have an important role in the control of complex interactions between the host and the microbiota, because those cells express receptors that are able to recognize selectively specific microbial patterns. The most characterized receptors are the TLR that are activated by the nuclear factor-*κ*B (NF-*κ*B) system and by production of cytokines, chemokines, and effectors of innate immunity [12, 13]. TLRs are able to recognize the characteristic structures of bacteria and viruses. For example, TLR-4 is essential in recognizing lipopolysaccharides (LPS) [12, 13], TLR-5 is essential in recognizing bacterial flagellin [12, 13], and TLR-9 is important in recognizing CpG islands of DNA [12, 13].

It has been demonstrated that all TLRs are expressed in the human colon and small intestine, but their functions are not well known yet. The TLRs are able to trigger an immune response against bacteria but also play an important role in protecting intestine from generical damage. For example, experimental studies in hosts knockout for TLR9, TLR4, and TLR2 have shown increased susceptibility to the development of a colitis induced by dextran sodium sulfate (DSS) [12, 13], as well as the protective role against DSS colitis induced by agonists of TLR2 and TLR4 supplement [12, 13].

It would seem that some strains of the microbiota also have a modulatory effect on the immune system of the GALT (gut associated lymphoid tissue), increasing the functionality of innate immunity, activating the dendritic cells, and stimulating NK cells by direct cytochemical action by pathogens penetrating into mucosa [12]. The intestinal microbiota can also regulate the activity of regulatory T cells (T-REG) that produce immunomodulatory interleukin and have an anti-inflammatory action and can activate T cells. Finally, helper T cells release IL-17 and IL-22, which have protective effects against enteropathogens genes [14]. There is great evidence that some patients suffering from irritable bowel syndrome (IBS) have an activation of the mucosal immune system [14]. In fact, some recent data show a significant increase of gene expression of TLR-4 on the colonic mucosa of the IBS patients compared to healthy subjects, although it is less than that observed in the colonic mucosa of patients with ulcerative colitis [15]. Recently, Martínez et al. [16] showed alterations in the integrity of the jejunal mucosal barrier in patients with IBS with diarrhea (IBS-D) and in particular at the apex junctional complex with an increased expression of Caudina 2, with a reduced phosphorylation of occludin and increased expression of myosin kinase. These changes are correlated with the activation of mast cells and abdominal pain reported by patients. However, the microbiota present in the intestinal lumen and in stools is different than the microbiota on the superficial layer of mucus covering the epithelium [17]. The most represented bacterial strains detected in the superficial layer of mucus are* Lactobacilli*, which are widely used as probiotics, and the* Clostridia* [17]. Some recent studies have shown that supernatants from cultures of some of the strains living in the intestinal mucus have an anti-inflammatory activity [17, 18]. These soluble substances produced by microorganisms are called postbiotics [18]. The first postbiotic described, obtained from the culture of* Faecalibacterium prausnitzii*, shows an anti-inflammatory effect in experimental model of colitis [19]. Similar effects were observed in supernatants obtained from cultures of* Lactobacillus paracasei*, which opposes the inflammation of the human intestinal mucosa induced by* Salmonella* Typhimurium [20]. Finally, also the supernatant obtained from a culture of* Lactobacillus rhamnosus* opposes the muscular alterations of the human colon induced by LPS of a pathogen strain of* Escherichia coli* [20]. This effect is mediated by activation of TLR-2 on the myocytes membrane and plays a protective effect in cardiac fiber cells [21].

In conclusion, the mesenchymal cells (fibroblasts and myocytes) that are located, respectively, in the lamina propria and* muscularis mucosae* of the intestinal epithelium recognize bacterial antigens circulating using the Toll-like receptors expressed in their cell membranes. Probably the activation of some of them by the microbiota and/or its secretory products can activate an anti-inflammatory response in the same intestinal mesenchymal cells, which are able to oppose a preinflammatory insult resulting from an alteration of intestinal permeability.

## 6. Interaction between Innate Immunity, Microbiota, and Probiotics

Probiotics are defined as bacteria having beneficial effects on the host. As most of them are driven from the gut microbiota, understanding how probiotics interact with the host can clarify how the microbiota interacts with the host. The mechanisms of action of probiotics have recently been explored [22]. As the microbiota, probiotics can be classified as inflammatory or anti-inflammatory according to their capacity to stimulate immune and nonimmune cells [23]. Probiotics may help preserve intestinal homeostasis by modulating the immune response and inducing the development of T-REG cells [23–25]. Similar to the microbiota, probiotics can be classified as inflammatory or anti-inflammatory depending on their capacity to stimulate immune and nonimmune cells [23].

One mechanism of probiotic action has been proposed based on the hypothesis that Crohn's disease (CD) susceptibility is linked to a defective initial innate immune response [26]. It has been demonstrated that the probiotics mixture* VSL#3* can induce NF-*κ*B nuclear translocation in epithelial cells followed by release of TNF-*α* and that this correlates with reduced epithelial permeability and susceptibility to CD-like ileitis in the SAMP1/YitFc mice that spontaneously develop the disease [27].

Although unexpected, this observation is very interesting. It has been recently shown that TNF-*α* can stimulate epithelial cell proliferation, and this occurs only when, in combination with IFN-*γ*, TNF-*α* induces epithelial cell apoptosis [28]. Hence, it is possible that, by upregulating TNF-*α*, probiotics may participate in epithelial barrier regeneration. The interaction of inflammatory bacteria with epithelial cells may be beneficial to host by bacterial ability to simulate innate immunity that protects against chronic inflammation. However, the same bacteria cannot improve overt disease in mice [27], and it may be deleterious as shown in other systems by using inflammatory probiotics [23]. Schlee et al. found that the probiotic* E. coli* strain* E. coli* Nissle 1917 induces beta defensin 2 upregulation in Caco-2 cells only when flagellin activity is restored [29]. A similar result was obtained in healthy volunteers using a cocktail of two probiotic* E. coli* strains [30]. A 78% upregulation was evident after three weeks of treatment, while defensin levels in fecal samples were still significantly elevated 9 weeks after the end of the treatment, indicating a more long-lasting effect.

*Lactobacillus plantarum v299* is able to induce an increase in* Muc3* expression in the jejunum and ileum of rats. However, this effect was only evident when live, but not heat-killed, bacteria were administered [31]. This suggests that metabolic activity of the bacteria is necessary for this action. The same strain upregulates* Muc3* expression and secretion on* HT29* cells, while at the same time limits adherence of* E. coli* E2348/49 strain [32].

Probiotic-driven metabolic products (called postbiotics) have been shown to enhance barrier function in a number of cases [33]. Culture supernatant of* S. boulardii*, but not* S. cerevisiae*, was able to improve significantly the epithelial cells ability in obtaining wound healing and migration* in vitro* and* in vivo* by *α*2*β*1 integrin collagen receptors activation [34].

Probiotic derived polyphosphate is able to protect mice against DSS-induced colitis, acting on the integrin-p38 MAPK pathway and suppressing oxidant-induced intestinal permeability by preventing F-actin and E-cadherin degradation [35]. In another study, the authors identified the p40 molecule, produced and secreted by* Lactobacillus GG*, as the main mediator for ameliorating DSS and oxazolone-induced inflammation, through its binding to epidermal growth factor receptor (EGFR). EGFR activation by p40 was sufficient to reduce cytokine-induced intestinal epithelial cells (IEC) apoptosis* in vitro* and* in vivo*. Further, the authors succeeded in administering the p40 molecule specifically in the colon, where once again they observed a subsequent activation of EGFR. Providing p40 was therefore able to both prevent and cure DSS-induced colitis [36]. This effect has never been observed before for live probiotic strains, as most might exert strain-specific preventive actions but are of little help once inflammation is manifested [27–37].

Mileti et al. have observed that* L. paracasei* strain B21060 has preventive effect in the DSS colitis model, whereas the culture supernatant exerts a prominent anti-inflammatory effect on the explants from the same patients [23]. Although the active component has not yet been identified, the protective action of the supernatant might in part be linked to epithelial barrier strengthening. In fact Mileti et al. have shown that when preconditioned with supernatant, healthy colonic mucosa explants are significantly more resistant to* Salmonella* infection [23].

These studies show clearly that shielding the intestinal barrier and preventing IEC apoptosis can be an important regulatory mechanism, which can influence the course of etiopathogenetic events both at the onset and during disease.

## 7. Conclusions

Understanding the relationship between intestinal microbiota and intestinal epithelium has increased our knowledge on pathophysiological conditions in the gastrointestinal and extraintestinal diseases.

Intestinal microbiota shows therefore a significant adaptation to different environmental stimuli. Microbiota is mandatory for several activities, ranging from the growth of the immune system to the synthesis of some amino acids and vitamins. It is hypothesized that the vast majority of those activities is mediated by diet and that changing in microbiota composition may permit the adaptation to host's metabolic and immunologic activities according to environmental changes. When this partnership is impaired, we have a significant risk to develop a disease. It seems to be particularly true not only for the occurrence of IBS, which affects large percentages of western population, but also for the occurrence of severe, disabling diseases, such as IBD.

Quali-quantitative changing in bacterial strains suggests that intestinal microbiota may be a therapeutic target based on “good” bacteria, called “probiotics,” and on probiotic-driven metabolic products, called “postbiotics.” New therapeutic approaches based on probiotics are now available, and further treatments based on postbiotics will come in the future.
